# Disease Severity and Prognosis of SARS-CoV-2 Infection in Hospitalized Patients Is Not Associated With Viral Load in Nasopharyngeal Swab

**DOI:** 10.3389/fmed.2021.714221

**Published:** 2021-09-10

**Authors:** Elisabetta Cocconcelli, Gioele Castelli, Francesco Onelia, Enrico Lavezzo, Chiara Giraudo, Nicol Bernardinello, Giulia Fichera, Davide Leoni, Marco Trevenzoli, Marina Saetta, Annamaria Cattelan, Andrea Crisanti, Paolo Spagnolo, Elisabetta Balestro

**Affiliations:** ^1^Department of Cardiac, Thoracic, Vascular Sciences and Public Health, University of Padova and Padova City Hospital, Padova, Italy; ^2^Department of Molecular Medicine, University of Padova and Padova City Hospital, Padova, Italy; ^3^Department of Medicine, Institute of Radiology, University of Padova and Padova City Hospital, Padova, Italy; ^4^Division of Infectious and Tropical Diseases, University of Padova and Padova City Hospital, Padova, Italy

**Keywords:** SARS-CoV-2, coronavirus disease 2019, viral load, hospitalized patients, outcome assessment

## Abstract

**Background:** The impact of viral burden on severity and prognosis of patients hospitalized for Coronavirus Disease 2019 (COVID-19) is still a matter of debate due to controversial results. Herein, we sought to assess viral load in the nasopharyngeal swab and its association with severity score indexes and prognostic parameters.

**Methods:** We included 127 symptomatic patients and 21 asymptomatic subjects with a diagnosis of SARS-CoV-2 infection obtained by reverse transcription polymerase chain reaction and presence of cycle threshold. According to the level of care needed during hospitalization, the population was categorized as high-intensity (HIMC, *n* = 76) or low intensity medical care setting (LIMC, *n* = 51).

**Results:** Viral load did not differ among asymptomatic, LIMC, and HIMC SARS-CoV-2 positive patients [4.4 (2.9–5.3) vs. 4.8 (3.6–6.1) vs. 4.6 (3.9–5.7) log10 copies/ml, respectively; *p* = 0.31]. Similar results were observed when asymptomatic individuals were compared to hospitalized patients [4.4 (2.9–5.3) vs. 4.68 (3.8–5.9) log10 copies/ml; *p* = 0.13]. When the study population was divided in High (HVL, *n* = 64) and Low Viral Load (LVL, *n* = 63) group no differences were observed in disease severity at diagnosis. Furthermore, LVL and HVL groups did not differ with regard to duration of hospital stay, number of bacterial co-infections, need for high-intensity medical care and number of deaths. The viral load was not an independent risk factor for HIMC in an adjusted multivariate regression model (OR: 1.59; 95% CI: 0.46–5.55, *p* = 0.46).

**Conclusions:** Viral load at diagnosis is similar in asymptomatic and hospitalized patients and is not associated with either worse outcomes during hospitalization. SARS CoV-2 viral load might not be the right tool to assist clinicians in risk-stratifying hospitalized patients.

## Introduction

Severe acute respiratory syndrome-Coronavirus-2 (SARS-CoV-2) is the etiological agent of the second pandemic infection of the 3rd millennium, following the H1N1 influenza outbreak in 2009. This new virus, which causes Coronavirus-Disease-19 (COVID-19), rapidly spread from China, where the first cases were discovered in late December 2019. As of February 2021, COVID-19 has infected more than 6,000,000 people worldwide.

Epidemiological studies found that a large fraction of individuals infected with SARS-CoV-2 are asymptomatic (1, 2). Yet, the greatest health care burden is accounted for by symptomatic patients. In this regard, COVID-19 may cause a wide range of clinical manifestations, ranging from mild flu-like symptoms with cough and fatigue to severe respiratory failure, leading to non-invasive/invasive mechanical ventilation (NIV/IMV) in the high-intensity (HIMC) or intensive medical care units (ICUs) (3).

Several studies tried to identify prognostic tools. Of these, chest X-rays (CXRs) at admission (4, 5), laboratory findings (6), and clinical composed scores (7) have been proposed as predictors of worst clinical outcomes.

The importance of COVID-19 viral load detectable in the nasopharyngeal swab has been addressed in a number of studies, yet with controversial results (8–11). In particular, it has been reported that the viral load reaches a peak during the first week from symptoms onset, followed by a decrease in the next 1 or 3 weeks. Others have described an independent association between the viral load and mortality or ICU admission (12–15). Conversely, Argyropoulos et al. did not find any associations between viral load and predictors of worst prognosis (i.e., admission to ICU, duration of oxygen supplementation and overall survival) (16). Similarly, other authors did not find any differences in the viral load between asymptomatic and symptomatic patients (1, 17). Finally, in France, patients from the summer outbreak displayed higher viral load with lower severity markers compared with patients from the spring outbreak (18).

With this background, we sought to assess the role of viral load, obtained from SARS-CoV-2 positive patients hospitalized in a tertiary care center in Padova, as a predictor of the need of High Intensity Medical Care (HIMC), and its relation with other established prognostic parameters.

## Materials and Methods

### Study Population and Study Design

Among subjects who were hospitalized for SARS-CoV-2 infection in the Division of Infectious and Tropical Diseases of the University Hospital of Padova between February and April 2020, we retrospectively collected 127 patients diagnosed by RT-PCR at nasopharyngeal swab (NP) and with the presence of Gene E cycle threshold (Ct) in the diagnostic RT-PCR. Were excluded patients whose sample was analyzed on a different diagnostic platform or at a different institution or with a different Gene Ct.

In our study population, demographical and clinical data, gas exchange values (PaO_2_/FiO_2_), blood samples, SARS-CoV-2 Gene E Ct, and chest X-rays (CXRs) were collected at hospital admission. Comorbidities were categorized as: cardiovascular diseases (CVDs), respiratory diseases, metabolic diseases (including diabetes mellitus, obesity, and dyslipidemia), autoimmune diseases and oncologic diseases (including lung, prostate, pancreatic, breast, and colon cancers). Twenty-one asymptomatic or mildly symptomatic SARS-CoV-2 positive patients treated at home, from the cohort previously reported by Lavezzo et al. (1) were included as controls.

Based on patient's clinical conditions during hospitalization, the study population was categorized according to the level of care needed. The use of high-flow nasal cannula (HFNC) or NIV/IMV which required admission either to the ICU or to the Respiratory ICU, was considered as a high-intensity medical care setting (HIMC, *n* = 76), while the need for oxygen supplementation through low-flow nasal cannula or face mask was considered as a low-intensity medical care setting (LIMC, *n* = 51), as previously described (5).

Moreover, in order to compare the clinical data according to the viral load, the overall study population was further categorized in two groups, namely High (HVL, *n* = 64) and Low Viral Load (LVL, *n* = 63).

### Radiological Evaluation

For each patient, a single image plane CXR was available at hospital admission. Two radiologists (C.G., G.B.) with more than 10-year experience in thoracic imaging, who were blind to clinical data, scored the images independently using a composite semi-quantitative scale, as previously described (4). Thus, a radiological global score (CARE) including ground-glass opacities and consolidations was assessed for each patient.

### SARS-CoV-2 Detection and Assessment of Genome Equivalents

Upper respiratory tract samples were collected by healthcare professionals with a flocked swab and immediately put into transport medium (eSwab, Copan Italia Spa). Sampling was performed either at the day of hospitalization or, at most, the day before for all patients. Detection of SARS-CoV-2 RNA was performed with an in-house reverse transcription polymerase chain reaction (RT–PCR) protocol, developed according to the diagnostic methodology by Corman et al. (19) with primers and probes targeting the gene encoding the envelope (E). Additionally, to assess the correct execution of the sampling, each sample was tested using primers designed to amplify the human housekeeping gene encoding RNase P, serving as an internal control. Reactions that failed to show the internal positive control were repeated. Ct data from real-time RT–PCR assays was collected for E gene. Genome equivalent copies per ml were inferred according to linear regression performed on calibration standard curves. The interpolated Ct values were further multiplied by 100, according to the final dilution factor (1:100). Linear regression was calculated in Python3.7.3 using modules scipy 1.4.1, numpy 1.18.1, and matplotlib 3.2.1.

### Ethics Statement

This was a retrospective study on anonymized patient's data collected from electronic medical records. The study protocol complies to the ethical guidelines of the 1975 Declaration of Helsinki and, in agreement with national regulation on retrospective observational studies, it was notified and approved by the local ethics committee (nr.: 46430/03.08.2020) and the need for patient's informed consent was waived.

### Statistical Analysis

Categorical variables were described as absolute (*n*) and relative values (%), whereas continuous variables were described as median and interquartile range. To compare demographic data and baseline clinical characteristics between asymptomatic, LIMC and HIMC groups or between LVL and HVL groups, Chi square test and Fisher's exact test for categorical variables and Kruskal-Wallis tests or Mann–Whitney *U*-test for continuous variables were used, as appropriate. The correlation was assessed using the non-parametric Spearman's rank method. In a univariate logistic regression analysis, followed by a regression model adjusted for gender, age, BMI, pack years, lag time symptoms—diagnosis, cardiovascular diseases, metabolic diseases, autoimmune diseases, oncologic diseases, respiratory diseases, we analyzed the role of viral load as predictor of the different level of care. All data were analyzed using SPSS Software version 25.0 (US: IBM Corp., New York, NY, USA). *p*-values < 0.05 were considered statistically significant.

## Results

### Viral Load Differences in Asymptomatic and Hospitalized Patients

Baseline demographic and viral load data of asymptomatic, LIMC, and HIMC SARS-CoV-2 positive patients included in the study are summarized in Table 1.

**Table 1:** Baseline demographics and viral load of SARS-CoV-2 positive asymptomatic patients and hospitalized patients for SARS-CoV-2 related infection *categorized in low* (LIMC) and *high* (HIMC) intensity medical care.

	**Asymptomatic patients ** **(*n* = 21)**	**Low-intensity medical care (LIMC) ** **(*n* = 51)**	**High-intensity medical care (HIMC) ** **(*n* = 76)**	***p*-value**
Male—*n (%)*	13 (62)	28 (55)	54 (71)	0.17
Age at diagnosis—*years*	65 (58–73)^*^	64 (52–75)^**^	77 (63–82)	**0.001**
BMI—*kg/m^2^*	24.9 (22.2–29.8)^*^	26.1 (21.2–29.2)^**^	30 (25–31)	**0.007**
Viral load (Gene E)—*log_10_ copies/ml*	4.4 (2.9–5.3)	4.8 (3.6–6.1)	4.6 (3.9–5.7)	0.31

*Values are expressed as numbers and (%) or median and interquartile range, as appropriate. To compare demographic Chi square test for categorical variables and Kruskal-Wallis for continuous variables were used.*

(*) *As compared to HIMC patients*

(**) *indicates the presence of statistically significant differences. Bold values are significant p-values*.

No differences in sex were observed across the study groups, although individuals were mostly males in each cohort (62 vs. 55 vs. 71%; respectively). Age and BMI were significantly different between both asymptomatic and LIMC patients as compared with HIMC patients [65 (58–73) vs. 64 (52–75) vs. 77 (63–82) years; *p* = 0.001 for age and 24.9 (22.2–29.8) vs. 26.1 (21.2–29.2) vs. 30 (25–31) kg/m^2^; *p* = 0.007, for BMI, respectively]. However, viral load did not differ across the three groups [4.4 (2.9–5.3) vs. 4.8 (3.6–6.1) vs. 4.6 (3.9–5.7) log_10_ copies/ml; *p* = 0.31] even when comparing asymptomatic individuals with all hospitalized patients [4.4 (2.9–5.3) vs. 4.68 (3.8–5.9) log_10_ copies/ml; *p* = 0.13].

### Patient Demographics and Clinical Characteristics at Baseline and During Hospitalization

Demographic and clinical characteristics of LIMC and HIMC group at admission and during hospitalization are summarized in Table 2.

**Table 2:** Baseline demographics and clinical features of the overall hospitalized study population for SARS-CoV-2 related infection, and of the two subgroups categorized in *low* (LIMC) and *high* (HIMC) intensity medical care.

	**Overall Hospitalized Study Population** ** (*n* = 127)**	**Low-intensity medical care (LIMC)** ** (*n* = 51)**	**High-intensity medical care (HIMC)** ** (*n* = 76)**	***p*-value**
Male—*n (%)*	82 (65)	28 (55)	54 (71)	0.06
Age at admission—*years*	72 (58–81)	64 (52–75)	77 (63–82)	**0.001**
Smoking history—*pack years*	0 (0–16)	0 (0–10)	0 (0–25)	0.29
Current—*n (%)*	6 (5)	3 (6)	3 (4)	0.61
Former—*n (%)*	54 (42)	19 (37)	35 (46)	0.32
Non-smokers—*n (%)*	67 (53)	29 (57)	38 (50)	0.52
BMI—kg/m^2^	27.1 (23.5–30.5)	26.1 (21.2–29.2)	30 (25–31)	**0.003**
Comorbidities—*n* (%)				
Cardiovascular diseases (CVD)	81 (64)	23 (45)	58 (76)	** <0.0001**
Chronic respiratory diseases	23 (18)	5 (10)	18 (24)	**0.04**
Autoimmune diseases	15 (12)	6 (12)	9 (12)	0.99
Metabolic diseases	54 (43)	14 (27)	40 (53)	**0.0003**
Oncologic diseases	20 (16)	8 (16)	12 (16)	0.98
Viral load (Gene E)—*log_10_ copies/ml*	4.68 (3.8–5.9)	4.8 (3.6–6.1)	4.6 (3.9–5.7)	0.96
Lag time symptoms–diagnosis—*days*	5 (1–7)	4 (0–7)	5 (2–7)	0.20
P/F at admission—*ratio*	225 (108–429)	429 (364–429)	125 (66–191)	** <0.0001**
CARE score at admission	7 (2–15)	3 (1–5)	13 (5–20)	** <0.0001**
Hospitalization—*days*	13 (5–24)	7 (3–13)	18 (8–29)	** <0.0001**
Bacterial co-infections—*n (%)*	40 (32)	7 (14)	33 (34)	** <0.0001**
Dead—*n (%)*	26 (20)	0 (0)	26 (34)	** <0.0001**

*Values are expressed as numbers and (%) or median and interquartile range, as appropriate. To compare demographic between LIMC and HIMC, Chi square test, and Fisher t-test (n <5) for categorical variables and Mann-Whitney t-test for continuous variables were used, as appropriate. Bold values are significant p-values*.

In the entire study population, most patients were males (65%) and the median age was 72 years. Half of them were non-smokers (53%) and the most prevalent comorbidities were CVDs (64%), followed by metabolic disease (49%).

According to the level of care required during hospitalization, 76 patients were classified as HIMC (when HFNC, or NIV or IVM were used) and 51 as LIMC (when low-flow nasal cannula or mask were used).

Compared to LIMC patients, HIMC patients were mainly males [71 (54%) vs. 28 (55%); *p* = 0.06], older [77 (63–82) vs. 64 (52–75) years; *p* = 0.001] and with a higher BMI [30 (25–31) vs. 26.1 (21.2–29.2) kg/m^2^; *p* = 0.003]. The HIMC and LIMC groups were similar with regard to smoking history. Regarding comorbidities, patients requiring HIMC had more frequently CVDs [58 (76%) vs. 23 (45%); *p* < 0.0001], metabolic diseases [40 (53%) vs. 14 (27%); *p* = 0.0003], and chronic respiratory diseases [18 (24%) vs. 5 (10%); *p* = 0.04], conversely, they did not differ for autoimmune and oncologic diseases.

The duration of symptoms before hospital admission did not differ between patients requiring HIMC and LIMC [5 (2–7) vs. 4 (0–7) days; *p* = 0.20]. At hospital admission, patients requiring HIMC displayed a higher impairment of respiratory gas exchange with a worse P/F ratio [125 (66–191) vs. 429 (364–429); *p* < 0.0001] and a higher CARE score [13 (5–20) vs. 3 (1–5); *p* < 0.0001]. HIMC patients presented also a longer duration of hospitalization [18 (8–29) vs. 7 (3–13) days; *p* < 0.0001], a higher number of bacterial co-infections [33 (34%) vs. 7 (14%); < 0.0001] and a worse outcome [26 (34%) of deaths vs. 0 (0%); *p* < 0.0001] compared to LIMC patients.

As previously mentioned, no differences were found in the viral load at the first positive nasopharyngeal swab between HIMC and LIMC patients [4.8 (3.6–6.1) vs. 4.6 (3.9–5.7) log_10_ copies/ml; *p* = 0.31].

### Comparison Between Patients With High and Low Viral Load

In further analysis, the study population was divided in two groups, namely High (HVL, *n* = 64) and Low Viral Load (LVL, *n* = 63), according to the median value of the viral load (i.e., 4.68 log_10_ copies/ml). Demographic and clinical characteristics at admission and during the hospitalization are summarized in Table 3.

**Table 3:** Baseline demographics and clinical features of the population hospitalized for SARS-CoV-2 related infection categorized in *low* (LVL) and *high* (HVL) viral load.

	**Low viral load (LVL)** ** (*n* = 63)**	**High viral load (HVL)** ** (*n* = 64)**	***p*-value**
Male—*n (%)*	40 (63)	42 (66)	0.80
Age at admission—*years*	74 (62–81)	70 (56–80)	0.19
Smoking history—*pack years*	1 (0–23)	0 (0–10)	0.17
Current—*n (%)*	2 (3)	4 (6)	0.41
Former—*n (%)*	28 (44)	20 (31)	0.12
Non-smokers—*n (%)*	20 (30)	32 (50)	**0.03**
BMI—*kg/m^2^*	29 (24.8–31.2)	26.1 (22.1–30)	**0.04**
Comorbidities—*n (%)*			
Cardiovascular diseases	47 (75)	34 (53)	**0.01**
Respiratory diseases	11 (17)	12 (19)	0.85
Autoimmune diseases	3 (5)	12 (19)	**0.02**
Metabolic diseases	34 (54)	20 (31)	**0.01**
Oncologic diseases	10 (16)	10 (16)	0.96
Lag time symptoms–diagnosis—*days*	5 (2–7)	4 (1–7)	0.12
PaO2/FiO2—ratio	209 (101–429)	283 (0–429)	0.52
CARE score at admission	9 (3–16)	5 (2–14)	0.20
High-intensity medical care—*n (%)*	39 (62)	37 (58)	0.64
Hospitalization—*days*	10 (6–24)	13 (4–22)	0.91
Bacterial co-infections—*n (%)*	19 (30)	21 (33)	0.65
Dead—*n (%)*	13 (21)	13 (20)	0.96
White cells count*-−10^9^/L*	6.2 (4.1–8.7)	5.1 (3.6–6.4)	0.07
Hemoglobin—*g/L*	132 (118–143)	129 (116–142)	0.71
Neutrophils—*10^9^/L*	4.8 (2.9–7.7)	3.7 (2.2–5.7)	**0.04**
Lymphocytes—*10^9^/L*	0.8 (0.6–1.2)	0.9 (0.5–1.2)	0.94
Monocytes—*10^9^/L*	0.4 (0.2–0.7)	0.4 (0.3–0.6)	0.71
Eosinophils—*10^9^/L*	0 (0–0.02)	0 (0–0.01)	0.69
C-reactive protein—*mg/dL*	109 (50–170)	61.5 (19–130)	**0.03**
D-Dimer—*μg/L*	299 (158–908)	217 (179–350)	0.06
Albumin—*g/L*	30 (26–33)	30.5 (27–36)	0.27
Ferritin- *μg/L*	876 (505–1,481)	849 (404–1,258)	0.64
LDH—*U/L*	341 (265–464)	282 (204–402)	**0.03**

*Values are expressed as numbers and (%) or median and interquartile range, as appropriate. To compare demographic between LVL and HVL, Chi square test and Fisher t-test (n <5) for categorical variables and Mann-Whitney t-test for continuous variables were used. LDH, Lactate Dehydrogenase; BMI, body mass index; CARE, radiological global score. Bold values are significant p-values*.

No differences in sex, age, smoking history, chronic respiratory diseases, and oncologic diseases were found between LVL and HVL. Compared to patients with LVLs, those with HVL included a higher percentage of non-smokers (50 vs. 30%; *p* = 0.03), and had a lower BMI [26.1 (22.1–30) vs. 29 (24.8–31.2) kg/m^2^; *p* = 0.04], more frequently autoimmune diseases [12 (19%) vs. 3 (5%); *p* = 0.02] and less frequently CVDs [34 (53%) vs. 47 (75%); *p* = 0.01] and metabolic disease [20 (31%) vs. 34 (54%); *p* = 0.01].

**Figure 1:**
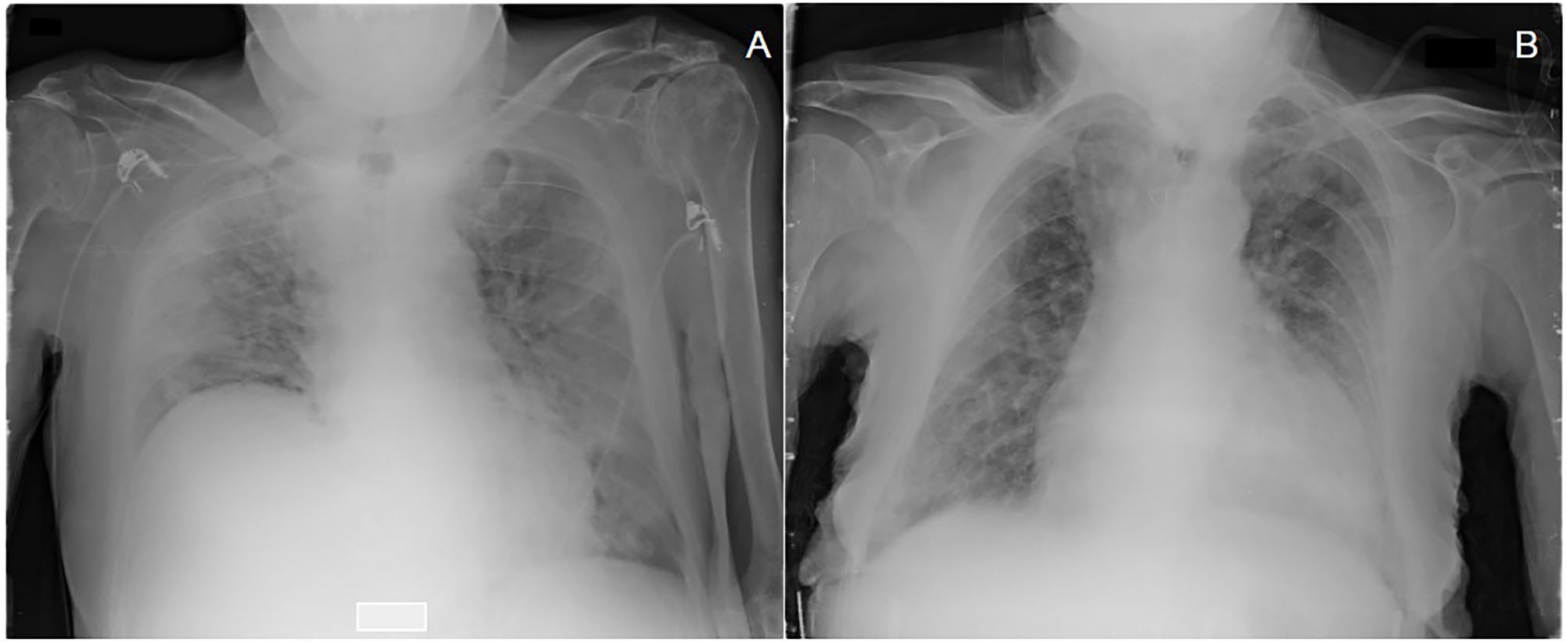
Chest X-ray with a high CARE score at admission of two patients treated with high intensity medical care (HIMC) during hospitalization and with different values of viral load (Gene E) at diagnosis: **(A)** a 81 years old patient presenting a CARE score of 22 points and a viral load of 2.43 log10 copies/ml (<25th percentile); **(B)** a 92 years old patient presenting a CARE score of 18 points and a viral load of 6.72 log10 copies/ml (>75th percentile).

Interestingly, disease severity at the emergency department was similar in the two groups regardless of viral load. In particular, patients with LVL and HVL showed the same CARE score, gas exchange impairment and symptom duration before diagnosis. Figure 1 displays the CXR of two patients with high CARE score requiring high intensity medical care but with different viral load at hospital admission (under 25th and over 75th interquartile, respectively). Blood samples at hospital admission revealed that neutrophils [3.7 (2.2–5.7) × 10^9^ vs. 4.8 (2.9–7.7) × 109/L; *p* = 0.04], C-reactive protein [61.5 (19–130) mg/dL vs. 109 (50–170) mg/dL; *p* = 0.03] and LDH [282 (204–402) U/L vs. 341 (265–464) U/L; *p* = 0.03] were lower in HVL compared to LVL. Of interest, LVL and HVL did not differ when considering other outcome measures such as duration of the hospital stay, number of bacterial co-infections, need for high-intensity medical care and number of deaths.

### Viral Load Correlations and Its Prognostic Role

A negative correlation between viral load at hospital admission and BMI was observed in the whole population (r = −0.26; *p* = 0.01). The viral load was also negatively correlated with the lag time (days) between symptoms initiation and diagnosis of SARS-CoV-2 infection by nasopharyngeal swab (r = −0.24; *p* = 0.007). The viral load was not an independent risk factor for HIMC in a univariate regression model (OR: 0.84; 95% CI 0.41–1.72, *p* = 0.64). This finding was confirmed when the regression model was adjusted for gender, age, BMI, pack years, lag time symptoms—diagnosis, and cardiovascular, metabolic, autoimmune, oncologic and respiratory diseases (OR: 1.59; 95% CI: 0.46–5.55, *p* = 0.46).

## Discussion

In this study, we explored the association between viral load detectable in the first positive nasopharyngeal swab and clinical outcomes of SARS-CoV-2 infected patients. Notably, the viral load did not differ between asymptomatic patients managed at home and patients who needed hospitalization. Moreover, when considering only hospitalized patients, viral load at first presentation was similar in patients requiring low intensity medical care (LIMC) and those requiring high-intensity (HIMC) setting. Moreover, viral load was not associated with either worse outcome measures during hospitalization or with mortality.

A large body of studies have addressed the issue of the viral load both in terms of change over time, from early detection of SARS-CoV-2 infection to recovery, and quantitative changes across the different respiratory samples (upper or lower respiratory tract) simultaneously collected. In particular, in a systematic review of 113 studies, Walsh et al. reported that the highest viral load from upper respiratory tract samples was observed at the time of symptoms onset and for a few days thereafter, with levels progressively slowing down over the following 1–3 weeks (8). Hence, in order to investigate the prognostic role of the viral load, we considered the first available nasopharyngeal swab positive for SARS-CoV-2 performed at admission and correlated it with clinical outcomes and prognosis. We observed that the viral load was similar across all study subsets of hospitalized (both LIMC and HIMC groups) and asymptomatic patients.

The association between viral load and disease severity remains controversial and debated. Previous studies have reported an association with severity of outcome. A retrospective cohort study of 875 patients hospitalized with COVID-19 in Brazil observed that SARS-CoV-2 viral load at admission was independently associated with mortality. However, the authors did not include comorbidities, clinical symptoms, and duration of symptoms before testing, which are clinically important variables and might have influenced the interpretation of their results (15). Similarly, Magleby et al. demonstrated that SARS-CoV-2 viral load at admission among hospitalized patients with COVID-19 (*n* = 678) independently correlates with the risk of intubation and in-hospital mortality. However, they reported a different symptom duration prior to admission between high and low viral load group (13). Pujadas et al. reported that the mean log_10_ viral load in patients who were alive (*n* = 807; mean log10 viral load 5.2 copies per mL [SD 3]) significantly differed from that of patients who died (*n* = 338; 6.4 copies per mL [2.7]). They also demonstrated an independent relationship between high viral load and mortality after adjusting for demographics and comorbidities (hazard ratio 1.07 [95% CI 1.03–1.11], *p* = 0.0014) (12).

In line with our findings, in a cohort of 205 patients from New York City, Argyropoulos et al. did not find any associations between viral load and clinical outcomes, including length of stay, oxygen support requirement, or survival (16), suggesting that severe symptoms and outcomes are unlikely to be related to high viral titers. However, this study evaluated mainly non-hospitalized patients with the exception of a small subset of patients with severe COVID-19 infection. In this regard, our study that conducted mild vs. severe hospitalized patients (i.e., LIMC and HIMC group) reinforces the lack of association between outcomes and viral load values. Notably, despite their similar viral load, these two populations differ in terms of age and BMI. Specifically, HIMC patients were older and had a significantly higher BMI compared to both asymptomatic and LIMC patients, in keeping with previous data from our group (5). If further confirmed, this finding is important, as predictors of worse outcome in individuals infected by SARS-CoV-2 are lacking.

In further analysis, we stratified patients requiring hospitalization based on their median viral load value (lower vs. higher). Age, sex, smoking history, and symptom duration before the diagnosis of COVID-19 were similar in patients with high (HVL) and low viral load (LVL). Viral load of SARS-CoV-2 peaks around the time of symptom onset (or a few days after) and decreases over time, and symptom duration negatively correlates with viral load. Of note, both the high and low viral load groups exhibited similar disease severity at diagnosis/admission, as assessed by PaO_2_/FiO_2_ ratio and radiographic CARE score, need for HIMC, number of bacterial co-infections, duration of hospitalization, and number of deaths. Similarly, the viral load was not associated with the need for high-intensity medical care both on univariate regression model and after adjusting for age, sex, BMI, pack years, symptoms duration, and cardiovascular, metabolic, autoimmune, oncologic, and respiratory comorbidities.

Our findings are in contrast with previous data (12, 13) suggesting that viral load is associated with mortality after adjustment for other concurrent clinical confounding factors. However, we analyzed not only the association of viral load with mortality, but also with the need for high-intensity medical care, which is associated with poor outcomes even long term. The lack of association between viral load and clinical outcomes and prognosis might suggest that the viral load in nasopharyngeal swab does not reflect the viral load in the lung and thus the severity of lung involvement and/or the degree of cytokine storm in the lung. In this regard, the level of a number of inflammatory markers (i.e., neutrophils, LDH, C-reactive-protein) is significantly increased in patients with a lower viral load.

The results of our study should be interpreted in light of some limitations, mainly the relatively small sample size, which implies that our findings need further validation in larger, independent, prospectively collected populations of patients. However, in contrast with previous studies, our patient population included a wide range of disease severity. Secondly, the timing of sampling could lead to a bias in viral load evaluation caused by different duration of disease. However, during the period evaluated by this study, our health care system centralized the patients to ER at the beginning of the disease, leading to a short duration of symptoms before diagnosis and viral load assessment: 5 days in overall population with no differences between LIMC and HIMC or LVL and HVL patients. Third, we retrospectively collected all clinical data from our electronic medical records; however, every effort was made to limit inaccuracy and missing data to a minimum. Finally, the assessment of viral load was performed on data coming from RT-PCR, whereas other methods would have been more appropriate to calculate the number of viral genome copies in a sample, such as digital PCR. The main problem of RT-PCR is that the relationship between viral load and Ct is not linear, with the former being extremely variable especially when the Ct is higher than 33 or 34. However, when Ct values are below this threshold, RT-PCR is a good proxy for viral load (20, 21).

The present study is retrospective and it has been performed on data collected from the diagnostic laboratory, where RT-PCR is the reference method for detecting SARS-CoV-2 infections (20). In addition, only 7 samples out of 127 (5.5%) in the present cohort have a Ct >33, thus accounting for a limited impact on the results.

In conclusion, this study shows that SARS-CoV-2 viral load at diagnosis is similar across asymptomatic patients, and patients hospitalized in a low and high intensity medical care. Moreover, viral load is not associated with either worse outcomes during hospitalization or with mortality. Therefore, SARS CoV-2 viral load assessed by RT-PCR might not be the most useful tool for patient risk stratification.

## Data Availability Statement

The raw data supporting the conclusions of this article will be made available by the authors, without undue reservation.

## Ethics Statement

The studies involving human participants were reviewed and approved by Comitato Etico per la Sperimentazione Clinica della Provincia di Padova. Written informed consent for participation was not required for this study in accordance with the national legislation and the institutional requirements. Written informed consent was obtained from the individual(s) for the publication of any potentially identifiable images or data included in this article.

## Author Contributions

EB, EC, GC, and EL conceived the study. EC coordinated data collection, curation, and analyses. EL, FO, and ACr coordinated laboratory testing on swabs and validated the results. EC, GC, and NB analyzed the data. EC, GC, NB, FO, DL, and MT performed data collection, direct contacting of study participants at follow up, and consistency check on metadata. CG and GF scored radiography. EC, GC, and EB wrote the manuscript with revision and supervision from PS, MS, and ACa. All authors contributed to the article and approved the submitted version.

## Conflict of Interest

The authors declare that the research was conducted in the absence of any commercial or financial relationships that could be construed as a potential conflict of interest.

## Publisher's Note

All claims expressed in this article are solely those of the authors and do not necessarily represent those of their affiliated organizations, or those of the publisher, the editors and the reviewers. Any product that may be evaluated in this article, or claim that may be made by its manufacturer, is not guaranteed or endorsed by the publisher.
